# Two Cases of Fracture of the Skull

**Published:** 1862-12

**Authors:** Hiram Wanzer

**Affiliations:** Chicago, Ill.


### ARTICLE XLII.

### TWO CASES OF FRACTURE OF THE SKULL.

By HIRAM WANZER, M.D., Chicago, Ill.

CASE 1.—Miss S., aged 22 years, of good constitution, was thrown from a carriage, receiving a compound comminuted fracture of the anterior inferior angle of the right parietal bone, over an area of about two inches; the pieces were depressed, so that their outer surfaces corresponded with the inner edge of the undepressed bone. The dura mater was entire. The patient was comatose, and suffering from shock. Upon removing the depressed bones, eight hours after the injury, consciousness was restored, and reaction began. Not fifteen minutes afterward, she was again comatose; and upon examination, the dura mater was found to be bulging outwards; and that protrusion steadily increased, till the membrane was slightly above the level of the cranium.

In accordance with what seems to be the weight of surgical authority, no effort was made to evacuate the extravasated fluid.

Death occurred twenty-four hours after the injury. The effusion under the membranes seemed to occur as a consequence of the removal of the pieces of bone which may have acted as a compress upon a wounded artery. Possibly, the patient might have been benefitted by incision of the dura mater; had this practice been followed, ligation of the external carotid might have been justifiable; seeing that death was threatened by hemorrhage, *probably* from laceration of the middle meningeal artery within the arachnoid sac.

CASE 2.—Charles C., aged 30, in February, '62, fell from the top of a freight car, striking upon the right side of the head. The body was cold, the pulse nearly imperceptible; there was coma and general paralysis. Blood was vomited continually, and in considerable quantities. Several drops of blood had run from the right ear, and at each effort at emesis a few drops were ejected from the meatus. There was no oozing of serous fluid. The usual restorative measures were used, and in about three hours reaction was established, and consciousness and motion restored. In all respects the case progressed favorably for one week; at the expiration of this time, decided symptoms of encephalitis set in. Bleeding to syncope, at once, and completely, removed this danger. The next week, there was paralysis of the muscles of the right side of the face; this symptom, coming on gradually, disappeared in a few days, leaving no evidence of the injury, excepting serious deafness in the right ear, with a sense of prickling in the meatus, and tenderness of the concha. The getting of blood from the ear, I am disposed to attribute to the congestion of the cranial cavity, occasioned by the act of vomiting. There was, probably, fracture of the petrous portion of the temporal bone, involving the membrana tympani, and more or less immediately the portio dura.

The source of the blood thrown from the stomach is a matter for conjecture.

